# Knowledge, attitudes, and decision-making regarding hyperbaric oxygen-assisted cancer treatment among related healthcare professionals

**DOI:** 10.3389/fonc.2026.1732828

**Published:** 2026-02-09

**Authors:** Cheng Zhou, Jia Zhang, Zhengze Dai, Dalin Fu, Yi Sun, Guopei Wu, Linsheng Chen, Xiao Lu

**Affiliations:** 1 Department of Rehabilitation, Jiangsu Province People’s Hospital and Nanjing Medical University First Affiliated Hospital, School of Rehabilitation Medicine, Nanjing Medical University, Nanjing, China; 2 Department of Nursing of Jiangsu Province People’s Hospital and Nanjing Medical University First Affiliated Hospital, Nanjing, China; 3 School of Rehabilitation Medicine, Nanjing Medical, Nanjing, China; 4 Department of Neurology, The Fourth Affiliated Hospital of Nanjing Medical University, Nanjing, China; 5 Department of Rehabilitation, Children’s Hospital of Nanjing Medical University, Nanjing, Jiangsu, China; 6 Department of Rehabilitation Medicine, Nantong Rici Hospital Affiliated to Yangzhou University, Nantong, Jiangsu, China

**Keywords:** cross-sectional study, decision making, hyperbaric oxygenation, knowledge, attitude, practice, neoplasms

## Abstract

**Aims:**

This study aimed to evaluate the knowledge, attitudes, and clinical decision-making of healthcare professionals in hyperbaric oxygen therapy (HBOT) units and oncology specialties regarding HBOT as an adjunctive cancer treatment.

**Methods:**

A cross-sectional survey was conducted between February, 2024 and February, 2025. Data were collected using a structured questionnaire, which included sections on demographic characteristics and assessed participants’ knowledge, attitudes, and practices (KAP) related to hyperbaric oxygen-assisted cancer therapy. The KAP scores were calculated to quantify the respondents’ familiarity with and perspectives on the treatment.

**Results:**

Of 202 valid questionnaires, 58.91% were physicians. The majority (63.86%) had over 15 years of clinical experience, and 79.70% reported prior HBOT training in oncology. The mean scores were 52.87 ± 12.21 for knowledge, 41.24 ± 5.92 for attitudes, and 22.40 ± 2.38 for decision-making. Analysis indicated that knowledge positively influenced attitudes (β=0.393, P = 0.013) and decision-making (β = 0.159, P = 0.018), while attitudes significantly impacted decision-making (β = 0.318, P = 0.012). Knowledge indirectly affected decision-making via attitude (β = 0.125, P = 0.004).

**Conclusions:**

Healthcare professionals working in HBOT units and oncology-related specialties demonstrated a generally adequate level of knowledge, positive attitudes, and a proactive approach toward the use of HBOT as an adjunctive cancer treatment, with attitude emerging as a key mediator linking knowledge to clinical decision-making. These findings highlight the importance of targeted educational interventions aimed at strengthening both knowledge and attitudes, which may in turn enhance evidence-based clinical decision-making and support the broader integration of HBOT into oncologic care pathways.

## Introduction

Hyperbaric oxygen therapy (HBOT) has evolved from its initial applications in decompression sickness to become an established treatment modality for diverse medical conditions. Hyperbaric oxygen therapy (HBOT) involves the administration of near-100% oxygen at pressures exceeding atmospheric pressure, typically ≥1.5 atmospheres absolute (ATA), in a specialized chamber, in accordance with definitions from international hyperbaric medicine societies; exposures below 1.5 ATA are generally classified as mild hyperbaric oxygen therapy and are not considered conventional HBOT (1). The therapy has gained recognition for its efficacy in treating conditions characterized by tissue hypoxia and impaired wound healing, with well-documented benefits in radiation-induced tissue injury, diabetic wounds, and certain infections (2).

Despite advances in conventional cancer treatments, including surgery, chemotherapy, and radiotherapy, significant challenges persist in managing radiation-induced toxicities that can severely impact patients’ quality of life. Approximately 50% of cancer patients undergo radiotherapy as part of their treatment, and among long-term survivors, 5-15% respectively experience serious radiation-related complications months or years after treatment (3, 4). These late radiation tissue injuries (LRTI) manifest as progressive deterioration characterized by reduced microvasculature, chronic inflammation, and fibrosis, eventually resulting in insufficient oxygen supply to maintain normal tissue function. Beyond its established role in alleviating radiation-induced tissue injury, hyperbaric oxygen therapy (HBOT) has also been explored for its potential to enhance the effects of conventional cancer treatments. Evidence supporting the use of HBOT in combination with radiotherapy is relatively consistent and reassuring, particularly in the management of radiation-induced toxicities and in improving tissue oxygenation following irradiation. In contrast, data regarding the interaction between HBOT and chemotherapy are less robust and remain heterogeneous, with studies reporting variable and sometimes conflicting findings depending on tumor type, treatment regimen, and timing of exposure (5). Therefore, while HBOT is widely regarded as a safe and effective adjunct in the context of radiotherapy-related complications, its role alongside chemotherapy warrants cautious interpretation and further investigation. The theory of KAP (Knowledge, Attitude and Practice) shows that knowledge is the basis of behavior change, while attitude and belief are the motive force of behavior change (6). As stated by KAP theory, the process of human behavior change can be divided into three steps: acquiring knowledge, generating attitudes/beliefs, and forming practice/behaviors (7). In the context of HBOT implementation, healthcare professionals’ knowledge about its mechanisms, especially benefits in radiation-induced tissue damage constitutes the foundation for their clinical decision-making. However, cognitive change caused by knowledge acquisition does not necessarily lead to behavior change; it should first lead to a change in perception, and then change people’s behavior through the change in perception (8). This theoretical framework is particularly relevant when examining how healthcare providers’ understanding of HBOT influences their decisions to integrate it into treatment protocols for cancers.

Healthcare professionals play a pivotal role in clinical decision-making regarding HBOT implementation, yet research examining their knowledge, attitudes, and practices in this domain remains limited, particularly in China. The challenges in implementing HBOT include availability of facilities, provider awareness of appropriate indications, and integration into standard treatment pathways (2). Our study aims to assess the knowledge, attitudes, and decision-making of healthcare professionals in China regarding HBOT for radiation-induced toxicities and its other applications in oncology, such as sensitizing chemotherapy and radiotherapy, and improving tumor oxygenation. This research will identify potential barriers to implementation and opportunities for educational interventions. It will provide valuable insights to optimize the integration of HBOT into comprehensive cancer treatment pathways, potentially improving the efficacy of tumor treatment and the prognosis of patients with radiation-induced tissue injuries.

## Materials and methods

### Study design and participants

This cross-sectional study was conducted between February, 2024 and February, 2025 at Jiangsu Provincial People’s Hospital, targeting healthcare professionals engaged in HBOT and anti-tumor treatment. Participants were eligible for inclusion if they met the following criteria (1): healthcare technicians who work on hyperbaric oxygen therapy (HBOT) or oncology-related clinical/scientific work (2); able to understand and complete questionnaires in Chinese. The study protocol was reviewed and approved by the Clinical Research Management Committee of Jiangsu Provincial People’s Hospital. Written Informed consent was obtained from all participants prior to data collection. Data were collected using Wenjuanxing, a widely used online survey platform in China, as well as on-site electronic data collection when necessary. Standardized administration of the questionnaire was ensured through uniform instructions provided to all participants, identical questionnaire content across collection modes, and consistent inclusion and exclusion criteria applied during data screening. Participants were invited to participate through institutional communication channels within the participating departments, including direct invitations during departmental meetings and electronic notifications distributed by department coordinators. All participants were treated as survey respondents rather than clinical cases. The questionnaire was self-administered in an anonymous format and completed either online via the Wenjuanxing platform or on-site using electronic devices. The survey was applied by the research team, who provided standardized instructions before participation. Participants were allowed to complete the questionnaire at their convenience within a predefined data collection period, and no strict time limit was imposed; however, response duration was monitored to ensure data quality.

### Questionnaire introduction

The final version of the questionnaire, administered in Chinese, consisted of four sections: demographic characteristics, knowledge, attitude, and decision-making. The complete questionnaire is provided as **Supplementary Material**. The knowledge section included 14 items, the attitude section 10 items, and the decision-making section 7 items, all measured using 5-point Likert scales. All questionnaire items were closed-ended, generating ordinal quantitative data rather than qualitative responses. The knowledge dimension assessed participants’ self-reported awareness and understanding of the principles, clinical indications, mechanisms, safety, and applications of HBOT in oncology. The attitude dimension evaluated participants’ beliefs, perceptions, and value judgments regarding the safety, effectiveness, cost-effectiveness, and collaborative potential of HBOT as an adjunctive cancer treatment. The decision-making dimension focused on participants’ stated clinical preferences and intentions concerning whether and under what conditions they would recommend or apply HBOT in cancer-related clinical scenarios. Knowledge scores ranged from 14 to 70, attitude scores from 10 to 50, and decision-making scores from 7 to 35. For all three dimensions, adequate knowledge, positive attitudes, and proactive decision-making were defined as achieving more than 70% of the maximum possible scores (9). The questionnaire design and analytical framework were guided by the Knowledge–Attitude–Practice (KAP) theory, which conceptualizes knowledge acquisition as a prerequisite for attitude formation, and attitude as a key determinant influencing subsequent behavioral intention and decision-making.

A total of 202 completed questionnaires were included in the final analysis. The internal consistency of the entire instrument was high, with a Cronbach’s α coefficient of 0.927. The Kaiser-Meyer-Olkin (KMO) measure of sampling adequacy was 0.908, indicating good construct validity and suitability for further analysis.

### Statistical methods

Data analysis was performed using IBM SPSS Statistics version 27.0 (IBM Corp., Armonk, NY, USA), and structural equation modeling (SEM) was conducted using AMOS version 26.0 (IBM Corp., Armonk, NY, USA). Descriptive statistics were used to summarize participants’ demographic characteristics and scores for knowledge (K), attitude (A), and decision-making (P). Continuous variables were presented as mean and standard deviation (SD), while categorical variables and item-level responses were expressed as frequencies and percentages. Univariate analyses were conducted to examine differences in K, A, and P scores across demographic subgroups. For comparisons involving two groups, the Mann–Whitney U test was employed, while the Kruskal–Wallis H test was used for comparisons among three or more groups, as the KAP scores did not follow a normal distribution. Spearman’s rank correlation analysis was used to assess the associations among the knowledge, attitude, and decision-making dimensions. To further identify potential influencing factors of decision-making behavior, a binary logistic regression model was constructed using categorized decision-making outcomes as the dependent variable. In addition, a structural equation modeling (SEM) framework was established to explore the interrelationships among the K, A, and P domains. Model fit was evaluated using multiple indices, including the root mean square error of approximation (RMSEA), incremental fit index (IFI), Tucker–Lewis index (TLI), and comparative fit index (CFI), to ensure an adequate representation of the hypothesized relationships. All statistical tests were two-tailed, and a p-value of less than 0.05 was considered indicative of statistical significance.

## Results

### Demographic information on participants

Initially, a total of 312 questionnaires were collected in this study. The following cases were excluded: 1) 2 cases disagreed with the study; 2) 11 cases had response times of less than 90 seconds; 3) 97 cases involved healthcare professionals who were not engaged in anti-tumor treatment or hyperbaric oxygen chamber therapy; 4) 97 cases in which the baseline questions (Q4) and Question 5 were all answered “No,”, leaving 202 valid responses. The exclusion criteria based on response time and baseline questions were applied to ensure data quality and relevance. Questionnaires completed in less than 90 seconds were excluded because such a short response duration was considered insufficient for thoughtful reading and accurate completion of all items, indicating potential inattentive or random responding. In addition, baseline screening questions (Q4 and Q5) were designed to identify whether participants had any professional involvement or prior exposure to hyperbaric oxygen therapy or oncology-related practice. Questionnaires in which both baseline items were answered “No” were excluded, as these respondents lacked relevant background necessary to meaningfully evaluate knowledge, attitudes, and decision-making related to HBOT-assisted cancer treatment. Among them, 132 (65.35%) were female, 119 (58.91%) were physicians, 104 (51.49%) were at least 43 years old, 129 (63.86%) had worked for at least 15 years or more, 69 (34.16%) were engaged in research or work related to oncology treatment, 164 (81.19%) participants had experience in HBOT-related research or work, 148 (73.27%) had often recommended HBOT to patients, 161 (79.7%) had received relevant training. Knowledge scores varied significantly by gender (P = 0.017), age (P = 0.001), years of work experience (P < 0.001), research or work related to ‘oncology treatment’ (P = 0.006), research or work related to ‘HBOT’ (P < 0.001), job position (P < 0.001), professional title (P = 0.005), department (P < 0.001), recommendation for therapy (P < 0.001), and relevant training (P < 0.001). Attitude scores varied significantly by job position (P = 0.015), department (P = 0.020), recommendation for therapy (P = 0.006), and relevant training (P = 0.007). Practice scores varied significantly by age (P = 0.016), years of work experience (P = 0.003), research or work related to ‘HBOT’ (P = 0.010), recommendation for therapy (P = 0.021), and relevant training (P = 0.002) (**Table 1**). As shown in **Table 1**, higher knowledge, attitude, and decision-making scores were generally observed among participants who were older, had longer work experience, held physician positions or higher professional titles, worked in HBOT-related departments, had prior HBOT-related training, and more frequently recommended HBOT in clinical practice.

**Table 1:** Baseline characteristics.

Variables	N (%)	Knowledge, mean ± SD	*P*	Attitude, mean ± SD	*P*	Decision-making, mean ± SD	*P*
N=202		52.87 ± 12.21		41.24 ± 5.92		22.40 ± 2.38	
Gender			0.017		0.103		0.845
Male	70 (34.65)	55.74 ± 11.37		42.47 ± 5.24		22.23 ± 2.24	
Female	132 (65.35)	51.35 ± 12.40		40.59 ± 6.17		22.49 ± 2.45	
Age			0.001		0.636		0.016
<43	98 (48.51)	49.93 ± 12.79		40.84 ± 6.45		21.94 ± 1.86	
≥43	104 (51.49)	55.64 ± 10.99		41.63 ± 5.37		22.84 ± 2.72	
Years of work experience			<0.001		0.181		0.003
<15 years	73 (36.14)	47.68 ± 12.30		40.25 ± 6.37		21.82 ± 1.92	
≥15 years	129 (63.86)	55.81 ± 11.17		41.81 ± 5.59		22.73 ± 2.55	
Research or work related to ‘oncology treatment’			0.006		0.951		0.437
Yes	69 (34.16)	49.12 ± 14.47		41.17 ± 6.63		22.13 ± 1.69	
No	133 (65.84)	54.82 ± 10.38		41.28 ± 5.54		22.54 ± 2.66	
Research or work related to ‘hyperbaric oxygen therapy’			<0.001		0.065		0.010
Yes	164 (81.19)	56.02 ± 10.38		41.56 ± 6.13		22.57 ± 2.53	
No	38 (18.81)	39.29 ± 10.07		39.87 ± 4.73		21.66 ± 1.38	
Job position			<0.001		0.015		0.945
Physician	119 (58.91)	55.60 ± 11.74		42.19 ± 5.79		22.37 ± 2.56	
Nurse	68 (33.66)	49.79 ± 12.04		39.75 ± 6.18		22.47 ± 2.24	
Other	15 (7.43)	45.20 ± 10.58		40.47 ± 4.26		22.33 ± 1.40	
Professional title			0.005		0.202		0.930
Intermediate or less	113 (55.94)	50.78 ± 12.44		40.80 ± 5.99		22.35 ± 2.14	
Associate senior or above	89 (44.06)	55.53 ± 11.43		41.81 ± 5.81		22.46 ± 2.66	
Department			<0.001		0.020		0.094
Hyperbaric Oxygen Department or Hyperbaric Oxygen Therapy Room	106 (52.48)	57.06 ± 9.77		42.21 ± 5.73		22.58 ± 2.36	
Rehabilitation Department	43 (21.29)	52.51 ± 11.51		40.37 ± 5.50		22.35 ± 2.45	
Medical Oncology Department	25 (12.38)	40.64 ± 12.84		37.92 ± 7.49		21.72 ± 1.17	
Other	28 (13.86)	48.50 ± 12.58		41.89 ± 4.53		22.43 ± 3.05	
Type of hospital			0.352		0.238		0.208
Public level 3	150 (74.26)	52.29 ± 12.78		40.89 ± 6.31		22.33 ± 2.32	
Nonpublic level 3	52 (25.74)	54.56 ± 10.30		42.25 ± 4.52		22.62 ± 2.56	
Recommended hyperbaric oxygen therapy to patients			<0.001		0.006		0.021
Yes, frequently	148 (73.27)	56.97 ± 10.11		41.88 ± 6.36		22.61 ± 2.62	
Yes, occasionally	35 (17.33)	44.71 ± 9.17		40.20 ± 3.41		21.83 ± 1.25	
Never	19 (9.41)	35.95 ± 10.00		38.21 ± 4.85		21.79 ± 1.69	
Hospital provides hyperbaric oxygen therapy			0.085		0.169		0.549
Yes	185 (91.58)	53.29 ± 12.01		41.12 ± 5.94		22.36 ± 2.34	
No	13 (6.44)	51.15 ± 13.23		44.00 ± 5.21		23.23 ± 3.11	
Not sure	4 (1.98)	39.25 ± 12.69		38.00 ± 5.42		21.75 ± 0.50	
Training related to hyperbaric oxygen therapy			<0.001		0.007		0.002
Yes	161 (79.7)	56.39 ± 10.07		41.76 ± 6.06		22.60 ± 2.53	
No	41 (20.3)	39.07 ± 9.91		39.20 ± 4.88		21.61 ± 1.45	

### Knowledge, attitude, and decision-making

All detailed item-level response distributions for the knowledge, attitude, and decision-making dimensions are provided in the Supplementary Material (**Supplementary Tables S1**–**S3**). The knowledge, attitude, and decision-making scores were 52.87 ± 12.21 (possible range: 14-70), 41.24 ± 5.92 (possible range: 10-50), and 22.40 ± 2.38 (possible range: 7-35), respectively. The distribution of knowledge dimensions showed that the three questions with the lowest number of participants choosing the ‘Very aware’ option were ‘The effectiveness of HBOT as an adjunct to cancer treatment is related to the pressure level, duration, and frequency of the therapy.’ (K14) with 16.34%, ‘HBOT can effect mitochondrial oxidase activity, effect ATP synthesis, and alter the cell proliferation cycle, thereby inhibiting tumor growth.’ (K10) with 19.8%, and ‘The effectiveness of HBOT as an adjunct to cancer treatment depends on the type, location, staging, and grading of the tumor.’ (K13) with 19.8% (**Supplementary Table S1**).

The distribution of attitude dimensions showed that only 18.32% strongly agree that HBOT may help slow the progression of malignant tumors (A5). Meanwhile, only 21.29% strongly agree that HBOT as an adjunct to cancer treatment is safe (A1) and effective (A2). Further, only 21.29% strongly agree that the benefits of HBOT as an adjunct to cancer treatment outweigh the risks (A3) (**Supplementary Table S2**).

Responses to the decision-making dimension showed that 4.95% disagreed and 3.47% strongly disagreed that the equipment configuration would determine whether they recommend the therapy (P6), 5.94% disagreed and 2.97% strongly disagreed that the staffing configuration would determine whether they recommend the therapy (P7), and 2.97% disagreed and 2.48% strongly disagreed that they would recommend HBOT to patients and their families based on their knowledge and acceptance (P5) (**Supplementary Table S3**).

### Correlation analysis

In the correlation analysis, significant positive correlations were found between knowledge and attitude (r = 0.486, P < 0.001), knowledge and decision-making (r = 0.345, P < 0.001), as well as attitude and decision-making (r = 0.508, P < 0.001), respectively (**Table 2**).

**Table 2:** Correlation analysis.

Dimensions	Knowledge	Attitude	Decision-making
Knowledge	1		
Attitude	0.486 (P<0.001)	1	
Decision-making	0.345 (P<0.001)	0.508 (P<0.001)	1

### Univariate and multivariate analysis for decision-making

Detailed effect estimates, including odds ratios, standardized coefficients, and corresponding confidence intervals, are presented in **Tables 3**, **4**, which form the basis for the interpretation of independent associations and mediation effects reported below.

**Table 3:** Univariate and multivariate analysis for decision-making dimension.

Variables	Univariate logistic regression		Multivariate logistic regression	
	OR (95%CI)	P	OR (95%CI)	P
Knowledge score	1.064 (1.035-1.094)	<0.001	0.990 (0.951-1.031)	0.631
Attitude score	1.352 (1.237-1.477)	<0.001	1.361 (1.230-1.506)	<0.001
Gender				
Male	1.191 (0.661-2.147)	0.560		
Female	ref			
Age				
<43	0.827 (0.471-1.454)	0.510		
≥43	ref			
Years of work experience				
<15 years	0.619 (0.340-1.127)	0.116		
≥15 years	ref			
Research or work related to ‘oncology treatment’				
Yes	0.940 (0.519-1.704)	0.840		
No	ref			
Research or work related to ‘hyperbaric oxygen therapy’				
Yes	3.008 (1.301-6.958)	0.010	1.434 (0.196-10.509)	0.723
No	ref		ref	
Job position				
Physician	ref			
Nurse	0.974 (0.530-1.790)	0.933		
Other	0.986 (0.330-2.951)	0.980		
Professional title				
Intermediate or less	ref			
Associate senior or above	0.798 (0.452-1.410)	0.437		
Department				
Hyperbaric Oxygen Department or Hyperbaric Oxygen Therapy Room	ref		ref	
Rehabilitation Department	0.732 (0.356-1.505)	0.397	1.413 (0.564-3.543)	0.461
Medical Oncology Department	0.280 (0.098-0.801)	0.018	2.134 (0.249-18.291)	0.489
Other	0.531 (0.220-1.279)	0.158	0.797 (0.214-2.971)	0.736
Type of hospital				
Public level 3	0.715 (0.378-1.352)	0.302		
Nonpublic level 3	ref			
Recommended hyperbaric oxygen therapy to patients				
Yes, frequently	3.365 (1.066-10.620)	0.038	0.558 (0.088-3.545)	0.536
Yes, occasionally	0.938 (0.236-3.724)	0.927	0.346 (0.066-1.823)	0.211
Never	ref		ref	
Training related to hyperbaric oxygen therapy				
Yes	4.131 (1.730-9.866)	0.001	3.625 (0.573-22.912)	0.171
No	ref		ref	

Multivariate logistic regression showed that attitude score (OR = 1.361, 95% CI: [1.230-1.506], P < 0.001) was independently associated with proactive decision-making (**Table 3**).

### Structural equation model

**Figure 1:**
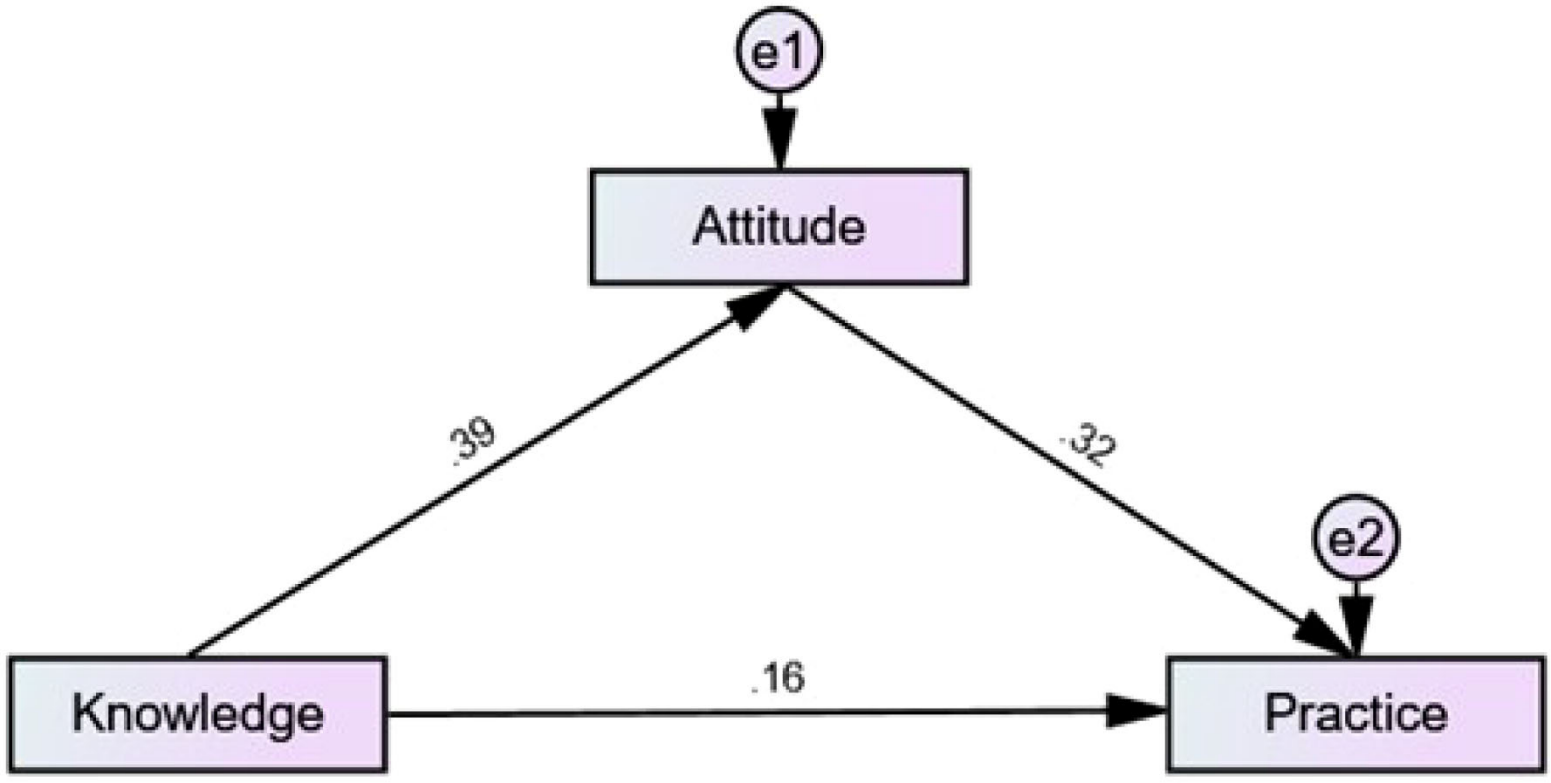
SEM model. The SEM framework was constructed based on the theoretical KAP model, with knowledge specified as an exogenous variable, attitude as a mediating variable, and decision-making as the outcome variable. The model demonstrates both direct and indirect pathways linking these domains, allowing simultaneous estimation of their interrelationships. The standardized path coefficients shown in **Figure 1** represent the strength and direction of the hypothesized associations, while the acceptable model fit indices indicate that the proposed structure adequately reflects the observed data.

Analysis of the direct and indirect effects of the model showed that the direct effect of knowledge on both attitude (β = 0.393, P = 0.013) and decision-making (β = 0.159, P = 0.018), as well as of attitude on decision-making (β = 0.318, P = 0.012), furthermore, knowledge indirectly affected decision-making through attitude (β = 0.125, P = 0.004) (**Table 4**; **Figure 1**).

**Table 4:** Direct and indirect effects.

Model paths	Standardized total effects		Standardized direct effects		Standardized indirect effects	
	β (95%CI)	P	β (95%CI)	P	β (95%CI)	P
Knowledge→Attitude	0.393 (0.193-0.570)	0.013	0.393 (0.193-0.570)	0.013		
Knowledge→Decision-making	0.284 (0.169-0.389)	0.010	0.159 (0.031-0.265)	0.018		
Attitude→Decision-making	0.318 (0.142-0.448)	0.012	0.318 (0.142-0.448)	0.012		
Knowledge→Decision-making					0.125 (0.072-0.216)	0.004

## Discussion

Healthcare professionals involved in HBOT and oncology-related fields demonstrated moderate to generally favorable levels of knowledge, attitudes, and decision-making regarding the use of HBOT as an adjunctive cancer treatment, with attitudes playing a key mediating role between knowledge and clinical practice. These findings underscore the need to incorporate targeted educational strategies that strengthen both knowledge and attitudes toward HBOT in oncology, thereby supporting more evidence-informed and confident clinical decision-making.

This study offers a detailed understanding of how healthcare professionals in HBOT units and oncology-related departments perceive and apply HBOT as an adjunctive cancer treatment. The findings revealed an overall pattern of moderate to favorable performance across knowledge, attitude, and decision-making dimensions, with significant interrelationships among the three. These results reflect a partially integrated KAP framework, in which knowledge appears to influence decision-making both directly and through its effect on attitude. Such a structure is consistent with previous KAP-based models, which emphasize that knowledge alone is not sufficient to change behavior without the reinforcement of favorable attitudes (10, 11). In our study, attitude emerged as a particularly important mediator, as supported by the structural equation modeling results showing both direct and indirect effects between knowledge, attitude, and decision-making.

The knowledge scores among participants were generally satisfactory, particularly with respect to basic concepts and commonly recognized clinical applications of HBOT. This finding is supported by the item-level results showing relatively higher proportions of “Very aware” responses for questions addressing the definition of HBOT, its traditional indications, safety profile, contraindications, and its established role in managing radiation-induced tissue injury (**Supplementary Table S1**, items K1–K5 and K7–K9). However, a closer examination of item-level distributions revealed uneven familiarity with more specialized or emerging aspects of HBOT, including its immunological mechanisms and its potential role in enhancing radiotherapy or chemotherapy sensitivity, as reflected by lower “Very aware” response rates for items related to mitochondrial effects, immune modulation, and treatment sensitization (**Supplementary Table S1**, items K10–K14). This pattern was particularly evident among participants from oncology departments, those with shorter work experience, and those without prior HBOT-related training, consistent with the subgroup differences reported in **Table 1**. Similar observations have been reported in previous studies, in which awareness of HBOT’s traditional indications is relatively widespread, whereas its emerging roles in oncology remain less familiar outside specialized hyperbaric settings (12, 13).

These knowledge disparities likely stem from the absence of structured educational integration of HBOT in most general oncology training programs. While continuing medical education often includes updates on drug therapies and surgical techniques, it tends to overlook adjunctive modalities such as HBOT, especially when they are perceived as outside the core domain of oncology (14, 15). In this context, it is important that hospitals and academic institutions incorporate updated, evidence-based content on HBOT into multidisciplinary training modules. For departments not directly involved in delivering HBOT, cross-departmental seminars, clinical case sharing, and shadowing opportunities in hyperbaric units may help bridge this knowledge divide. Without these measures, the potential clinical benefits of HBOT in cancer treatment may remain underutilized simply due to informational asymmetry.

Attitudes toward HBOT were generally favorable, with most respondents recognizing its safety, potential efficacy, and collaborative value across specialties. Nonetheless, hesitations were observed around its cost-effectiveness and long-term impact on tumor progression, reflecting uncertainty in areas where high-quality clinical evidence is still evolving. This cautious optimism is not unique to our setting; studies in similar healthcare systems have reported comparable patterns, in which enthusiasm about HBOT’s supportive role coexists with skepticism about its integration into standard oncology protocols (16, 17). Interestingly, training experience and departmental affiliation were associated with differences in attitude scores, as indicated by the subgroup comparisons reported in **Table 1**.

What is particularly noteworthy is that attitude, rather than knowledge alone, was the strongest and most consistent predictor of decision-making behavior in our multivariate analysis. This aligns with findings from health behavior research showing that professionals are more likely to implement what they feel positively about, especially in complex or interdisciplinary domains (18, 19). Given that attitudes are shaped not only by formal education but also by institutional support, professional discourse, and firsthand clinical outcomes, strategies to foster favorable attitudes must go beyond individual learning. Hospitals could organize interdisciplinary case discussions and patient outcome reviews involving both oncologists and hyperbaric specialists, thus reinforcing shared understanding and clinical trust. Additionally, institutions should provide clear administrative pathways and referral systems that legitimize the use of HBOT in appropriate oncology cases, thereby reinforcing its perceived value.

The decision-making practices observed in this study were mostly proactive, but they were also shaped by structural and contextual factors. Clinicians frequently cited patient condition, previous treatment response, and family preference as decision drivers, but they also reported that infrastructure, staffing, and equipment availability played important roles. This indicates that even well-informed and positively inclined professionals may be constrained by system-level limitations. Other studies in similar resource settings have echoed this concern, showing that decisions about non-standard treatments are often moderated by institutional readiness and perceived administrative support (20, 21). In our data, those working in facilities without dedicated HBOT services or with less robust staffing appeared less likely to recommend the therapy, despite having comparable knowledge or attitude scores.

To enable more consistent and confident decision-making, healthcare systems should invest in the operational integration of HBOT into cancer treatment workflows. This includes not only expanding access to treatment chambers and trained personnel, but also ensuring that clinical guidelines explicitly acknowledge HBOT as a viable option in certain oncology scenarios (22, 23). At a policy level, health authorities could develop funding models or reimbursement schemes that reflect the long-term cost-benefit profile of HBOT, particularly in managing radiation-related side effects where its efficacy is better established (24, 25). Furthermore, oncology departments could assign dedicated liaisons to work with hyperbaric units, ensuring smoother communication and patient transitions.

However, this study has several limitations that should be acknowledged. First, the cross-sectional design limits the ability to establish causal relationships between knowledge, attitudes, and decision-making behaviors. Second, the use of self-reported questionnaires may introduce response bias, as participants might overestimate their knowledge or provide socially desirable answers. Third, the sample was limited to a specific geographic region and professional group, which may affect the generalizability of the findings to broader healthcare populations. In addition, it should be noted that the overall level of clinical evidence supporting HBOT as an adjunctive treatment in oncology remains limited. Large-scale randomized controlled trials in this field are scarce, and in some areas largely absent, which partly explains the ongoing skepticism regarding the efficacy of HBOT in cancer treatment. Therefore, favorable attitudes toward HBOT should be viewed not as definitive confirmation of efficacy, but as a foundation for promoting well-designed, larger clinical trials aimed at generating stronger and more conclusive evidence. Future studies could also expand the administration of the questionnaire to international institutions and healthcare systems, which may help obtain a more diverse and representative sample and allow for cross-cultural comparisons in knowledge, attitudes, and decision-making regarding HBOT in oncology.

## Conclusion

In conclusion, the findings suggest that healthcare professionals in HBOT and oncology-related fields generally possess a solid understanding and hold favorable attitudes toward the use of HBOT in cancer treatment, with attitudes playing a mediating role between knowledge and clinical decision-making. To enhance evidence-based application of HBOT in oncology, targeted educational interventions that strengthen both knowledge and attitudes may further promote informed and proactive clinical decisions.

## Data Availability

The original contributions presented in the study are included in the article/**Supplementary Material**. Further inquiries can be directed to the corresponding authors.
