# Supplementary material for: Knowledge, attitudes, and decision-making regarding hyperbaric oxygen-assisted cancer treatment among related healthcare professionals
Source: Front Oncol. 2026 Feb 9;16:1732828. doi: 10.3389/fonc.2026.1732828 (PMC12926112; doi:10.3389/fonc.2026.1732828)
Supplement: Supplementary file 1 [file Table1.docx]

**Supplementary table 1. Distribution of knowledge dimension responses**

| **Knowledge** | **N (%)** | | | | |
| --- | --- | --- | --- | --- | --- |
|  | **Completely unaware** | **Slightly aware** | **Somewhat aware** | **Fairly aware** | **Very aware** |
| 1. Hyperbaric oxygen therapy is defined as: a treatment method where the human body breathes pure oxygen or high-concentration oxygen in an environment with a pressure higher than one atmosphere, equalized with the surrounding environment. | 9 (4.46) | 14 (6.93) | 29 (14.36) | 34 (16.83) | 116 (57.43) |
| 2. Hyperbaric oxygen therapy is mainly used for hypoxic, ischemic diseases, or diseases caused by ischemia or hypoxia, as well as a series of conditions related to ischemia and hypoxia during disease progression. | 3 (1.49) | 6 (2.97) | 29 (14.36) | 41 (20.3) | 123 (60.89) |
| 3. Hyperbaric oxygen therapy, as a treatment to improve ischemia and hypoxia in human tissues, organs, and cells, has been clinically used internationally and domestically for over 20 years. | 4 (1.98) | 9 (4.46) | 34 (16.83) | 48 (23.76) | 107 (52.97) |
| 4. According to the Chinese Medical Association Hyperbaric Oxygen Medicine Branch's "Consensus on Indications for Hyperbaric Oxygen Therapy (2018 version)": "Radiation tissue damage (including radiation osteonecrosis, soft tissue radiation necrosis, radiation hemorrhagic cystitis, radiation proctitis, preoperative and postoperative preventive treatment of radiation jaw damage in oral surgery)" is the class I indication for hyperbaric oxygen therapy, and "Adjunctive treatment for malignant tumors (used in conjunction with radiotherapy or chemotherapy)" is the class II indication for hyperbaric oxygen therapy. | 7 (3.47) | 27 (13.37) | 46 (22.77) | 48 (23.76) | 74 (36.63) |
| 5. Hyperbaric oxygen therapy can be used to reduce radiation tissue damage, such as radiation cystitis, radiation enteritis, and other radiation treatment injuries. | 9 (4.46) | 25 (12.38) | 47 (23.27) | 54 (26.73) | 67 (33.17) |
| 6. Hyperbaric oxygen therapy can be used as an adjunct to existing cancer treatments such as chemotherapy, radiotherapy, gene therapy, and photodynamic therapy, either before, after, or simultaneously with cancer treatments. | 8 (3.96) | 37 (18.32) | 49 (24.26) | 55 (27.23) | 53 (26.24) |
| 7. Hyperbaric oxygen therapy has been applied clinically for many years, with side effects including barotrauma, oxygen toxicity, and decompression sickness, all of which have a low incidence and are generally controllable. | 3 (1.49) | 11 (5.45) | 30 (14.85) | 51 (25.25) | 107 (52.97) |
| 8. Active pneumothorax and mediastinal emphysema are absolute contraindications for hyperbaric oxygen therapy. | 3 (1.49) | 6 (2.97) | 24 (11.88) | 40 (19.8) | 129 (63.86) |
| 9. Hyperbaric oxygen therapy can improve tissue hypoxia, including improving the hypoxic tumor microenvironment. | 2 (0.99) | 16 (7.92) | 39 (19.31) | 64 (31.68) | 81 (40.1) |
| 10. Hyperbaric oxygen therapy can reduce mitochondrial oxidase activity, decrease ATP synthesis, and alter the cell proliferation cycle, thereby inhibiting tumor growth. | 11 (5.45) | 43 (21.29) | 64 (31.68) | 44 (21.78) | 40 (19.8) |
| 11. Hyperbaric oxygen therapy can significantly enhance the activity of natural killer cells, nonspecific tumor-killing cells, and cytotoxic T lymphocytes, thereby improving the body’s immune response to tumors. | 7 (3.47) | 39 (19.31) | 62 (30.69) | 48 (23.76) | 46 (22.77) |
| 12. Hyperbaric oxygen therapy can increase cell membrane permeability, improve drug delivery efficiency, facilitate the entry of anti-cancer drugs into cells, and enhance cancer cell sensitivity to anti-cancer drugs. | 9 (4.46) | 42 (20.79) | 62 (30.69) | 46 (22.77) | 43 (21.29) |
| 13. The effectiveness of hyperbaric oxygen therapy as an adjunct to cancer treatment depends on the type, location, staging, and grading of the tumor. | 11 (5.45) | 55 (27.23) | 54 (26.73) | 42 (20.79) | 40 (19.8) |
| 14. The effectiveness of hyperbaric oxygen therapy as an adjunct to cancer treatment is related to the pressure level, duration, and frequency of the therapy. | 12 (5.94) | 54 (26.73) | 60 (29.7) | 43 (21.29) | 33 (16.34) |

**Supplementary table 2.Distribution of attitude dimension responses**

| **Attitude** | **N (%)** | | | | |
| --- | --- | --- | --- | --- | --- |
|  | **Strongly agree** | **Agree** | **Neutral** | **Disagree** | **Strongly disagree** |
| 1. I believe that hyperbaric oxygen therapy as an adjunct to cancer treatment is safe | 43 (21.29) | 98 (48.51) | 53 (26.24) | 5 (2.48) | 3 (1.49) |
| 2. I believe that hyperbaric oxygen therapy as an adjunct to cancer treatment is effective | 43 (21.29) | 103 (50.99) | 52 (25.74) | 3 (1.49) | 1 (0.5) |
| 3. I believe that the benefits of hyperbaric oxygen therapy as an adjunct to cancer treatment outweigh the risks | 43 (21.29) | 107 (52.97) | 48 (23.76) | 2 (0.99) | 2 (0.99) |
| 4. I believe that hyperbaric oxygen therapy is a relatively cost-effective adjunct treatment for cancer patients (Hyperbaric oxygen therapy generally involves one session per day, costing around 100 yuan per session, lasting about 1 hour, with 10 sessions forming one treatment course, depending on the situation). | 51 (25.25) | 103 (50.99) | 44 (21.78) | 2 (0.99) | 2 (0.99) |
| 5. I believe that hyperbaric oxygen therapy may help slow the progression of malignant tumors | 37 (18.32) | 90 (44.55) | 66 (32.67) | 6 (2.97) | 3 (1.49) |
| 6. I believe that hyperbaric oxygen therapy can effectively prevent and treat radiation-related adverse reactions in cancer patients | 55 (27.23) | 96 (47.52) | 44 (21.78) | 3 (1.49) | 4 (1.98) |
| 7. I believe that patients who require hyperbaric oxygen therapy should be evaluated and treated by qualified hyperbaric oxygen specialists, who should develop scientific and reasonable treatment plans and implement them | 121 (59.9) | 71 (35.15) | 7 (3.47) |  | 3 (1.49) |
| 8. I would like to receive professional knowledge and skills training related to hyperbaric oxygen therapy | 117 (57.92) | 73 (36.14) | 9 (4.46) | 1 (0.5) | 2 (0.99) |
| 9. I believe that oncologists can collaborate with hyperbaric oxygen specialists to conduct clinical work, and that it is a treatment method worth trying for cancer patients | 98 (48.51) | 94 (46.53) | 8 (3.96) |  | 2 (0.99) |
| 10. I believe that oncologists can collaborate with hyperbaric oxygen specialists to carry out basic and clinical research, which has strong innovation potential and clinical application prospects | 98 (48.51) | 94 (46.53) | 8 (3.96) |  | 2 (0.99) |

**Supplementary table 3. Distribution of decision-making dimension responses**

| **Decision-making** | **N (%)** | | | | |
| --- | --- | --- | --- | --- | --- |
|  | **Strongly agree** | **Agree** | **Neutral** | **Disagree** | **Strongly disagree** |
| 1. I would decide whether to recommend hyperbaric oxygen therapy based on the patient's tumor type, grade, and stage(P) | 62 (30.69) | 95 (47.03) | 34 (16.83) | 7 (3.47) | 4 (1.98) |
| 2. I would decide whether to recommend hyperbaric oxygen therapy based on the patient's response to chemotherapy and radiotherapy(P) | 66 (32.67) | 102 (50.5) | 25 (12.38) | 7 (3.47) | 2 (0.99) |
| 3. I would decide whether to recommend hyperbaric oxygen therapy based on the patient's overall health status and tolerance (P) | 76 (37.62) | 102 (50.5) | 18 (8.91) | 3 (1.49) | 3 (1.49) |
| 4. I would decide whether to recommend hyperbaric oxygen therapy based on the patient's financial situation(P) | 62 (30.69) | 108 (53.47) | 18 (8.91) | 12 (5.94) | 2 (0.99) |
| 5. I would decide whether to recommend hyperbaric oxygen therapy based on the patient's and their family's understanding and acceptance of the therapy(P) | 66 (32.67) | 108 (53.47) | 17 (8.42) | 6 (2.97) | 5 (2.48) |
| 6. The equipment configuration of the hyperbaric oxygen department in the hospital would determine whether I recommend hyperbaric oxygen therapy for cancer patients(P) | 56 (27.72) | 104 (51.49) | 25 (12.38) | 10 (4.95) | 7 (3.47) |
| 7. The staffing configuration of the hyperbaric oxygen department in the hospital would determine whether I recommend hyperbaric oxygen therapy for cancer patients (P) | 53 (26.24) | 107 (52.97) | 24 (11.88) | 12 (5.94) | 6 (2.97) |
